# Early impairments in the retina of rats fed with high fructose/high fat diet are associated with glucose metabolism deregulation but not dyslipidaemia

**DOI:** 10.1038/s41598-019-42528-9

**Published:** 2019-04-12

**Authors:** Elisa Vidal, Elise Lalarme, Marie-Annick Maire, Valérie Febvret, Stéphane Grégoire, Ségolène Gambert, Niyazi Acar, Lionel Bretillon

**Affiliations:** 1 Eye and Nutrition Research Group, Centre des Sciences du Goût et de l’Alimentation, AgroSup Dijon, INRA, CNRS, Université Bourgogne Franche-Comté, Dijon, France; 2 Horus Pharma Laboratories, Saint Laurent du Var, France; 3 Animalerie Expérimentale, Centre des Sciences du Goût et de l’Alimentation, AgroSup Dijon, INRA, CNRS, Université Bourgogne Franche-Comté, Dijon, France; 4 Clinical Biochemistry Department, University Hospital, Dijon, France

## Abstract

Way of life changes such as high consumption of processed foods rich in fat and sugar and sedentary lifestyle are associated with the increasing prevalence of metabolic syndrome (MetS) that affects about 35% in the American population. MetS is the main risk factor for diabetes mellitus, which is associated with vascular changes in the retina. However, the early consequences of MetS in the retina are not well described. We therefore aimed at characterizing the early effects of a high fructose and high fat diet (HFHF) on the function and structure of the rat retina, and evaluate the associations with metabolic changes. Brown Norway rats of 6 weeks of age were fed for 8 days, 5 weeks or 13 weeks with HFHF diet, or a standard chow. After only 4 weeks of this diet, rats exhibited a reduction in cone photoreceptor sensitivity to light. Moreover, we observed that MetS significantly exacerbated laser-induced choroidal neovascularization by 72% and 67% 2 weeks and 3 weeks post laser treatment, respectively. These retinal abnormalities were associated with deregulation of glucose metabolism but not lipid metabolism. These data showed retinal modifications in HFHF-induced MetS in the rat, at very early stage of the disease.

## Introduction

Metabolic syndrome (MetS) appears to be a risk factor for the development of Diabetic Retinopathy (DR)^1^ and Age-related Macular Degeneration (AMD)^2^, which are the leading causes of visual impairment in western populations before and after the age of 50 years, respectively^3^. Associated with lifestyle changes, i.e. low physical activity and high energy intake, the incidence of MetS is increasing worldwide^4^, reaching 20–25% of the adult population^5^. Met is characterized by a cluster of abnormalities like dyslipidaemia, hyperglycaemia, insulin resistance, hypertriglyceridaemia, hypertension, and an excess of abdominal fat^4^. Consumption of fructose via processed food and as sweetener is now well described to trigger metabolic disturbances like dyslipidaemia and hyperglycaemia^6,7^. Fructose has been very popular since the 1970s as a sweetener in the food industry because it is cheap to produce and is sweeter than most other sugars. Metabolic disturbances observed in fructose fed rats are divergent from one study to another because differences in study design^8^, animal strain^9^, duration of the diet, route of fructose administration^10^, age of the animals. Nevertheless feeding rodents with high fructose has been associated with visceral adiposity, dyslipidaemia and insulin resistance^11,12^, supporting the use of high fructose diet as a model to trigger metabolic syndrome. Only few animal models take into account the consequences on the retina of poor food quality^13^. Indeed, most studies use genetics or pharmacological drug administration to trigger microvascular alterations in the retina in connection to diabetes. To our knowledge, only one study showed that there are early modifications in the retina of rats fed with 60% of fructose^14^. The causes of the early consequences of visual impairment reported in MetS are nevertheless still under debate^15^.

It must be noticed that no study using fructose alone, including our^14^, recapitulated all the clinical features of MetS, including dyslipidaemia and hyperglycaemia on the one hand, and retinal alterations on the other hand.

In the present study, we hypothesized that a high fructose plus high fat diet would be sufficient to trigger MetS and retinal alterations in the rat. For that purpose, we characterized the consequences of feeding Brown Norway rats with a 60% Fructose – 9% saturated fat-diet in a time dependent manner on lipid and glucose metabolisms as well as on the functioning and development of vascular alterations in the retina.

## Results

### HFHF diet induced an increase in body fat but not in body weight

**Figure 1:**
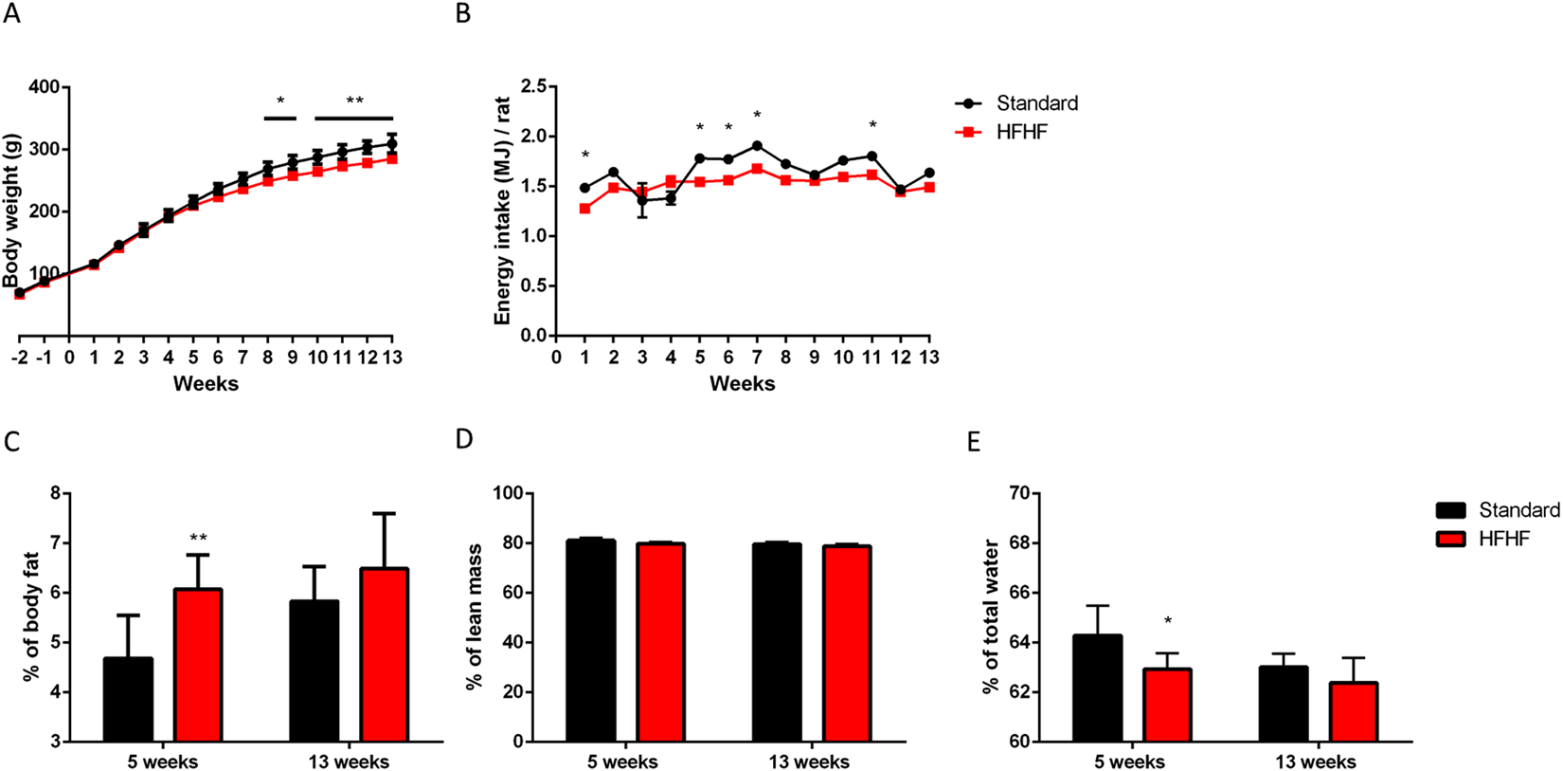
Effect of HFHF diet on body weight gain and tissues. (**A**) Body weight (grams) evolution and (**B**) energy intake (MJ/rat) during the 13 weeks of nutritional experiments. (**C**) Percentage of total body fat per rat. (**D**) Percentage of lean mass per rat. (**E**) Percentage of total water per rat. Figures A and B: Values are means ± SEM. ANOVA followed by Bonferroni test is performed. Figures C to E: Values are means ± SD (n = 8). Mann-Whitney test was performed. Data with different * are significantly different at P < 0.05; ** at P < 0.01; *** at P < 0.001.

Food consumption and rat’s body weight were recorded weekly during the entire experiment. As shown in Fig. 1A, standard fed rats had significantly higher body weight than HFHF diet fed rats from 8 weeks of diet until the end of the experiment (P < 0.05). Despite the difference, since HFHF provided +17% more energy than the standard diet (17.2 vs 14.74MJ/kg diet respectively), energy intake was similar in both groups of animals (Fig. 1B). A significantly higher food and water consumption was observed in rats fed with the standard diet compared to rats fed with HFHF diet (Supplementary Fig. 1, P < 0.05).

Body composition was measured by non-invasive EchoMRI. HFHF fed rats had larger body fat mass than rats from the standard diet at 5 weeks of diet (P < 0.05) but this effect was not persistent (Fig. 1C). Despite the percentage of lean mass did not significantly change between HFHF and standard groups (Fig. 1D), the percentage of total water marginally decreased in the HFHF fed rats at 5 weeks (Fig. 1E, P < 0.05).

### HFHF diet induced hyperglycaemia, glucose intolerance, insulin resistance, but not dyslipidaemia

**Figure 2:**
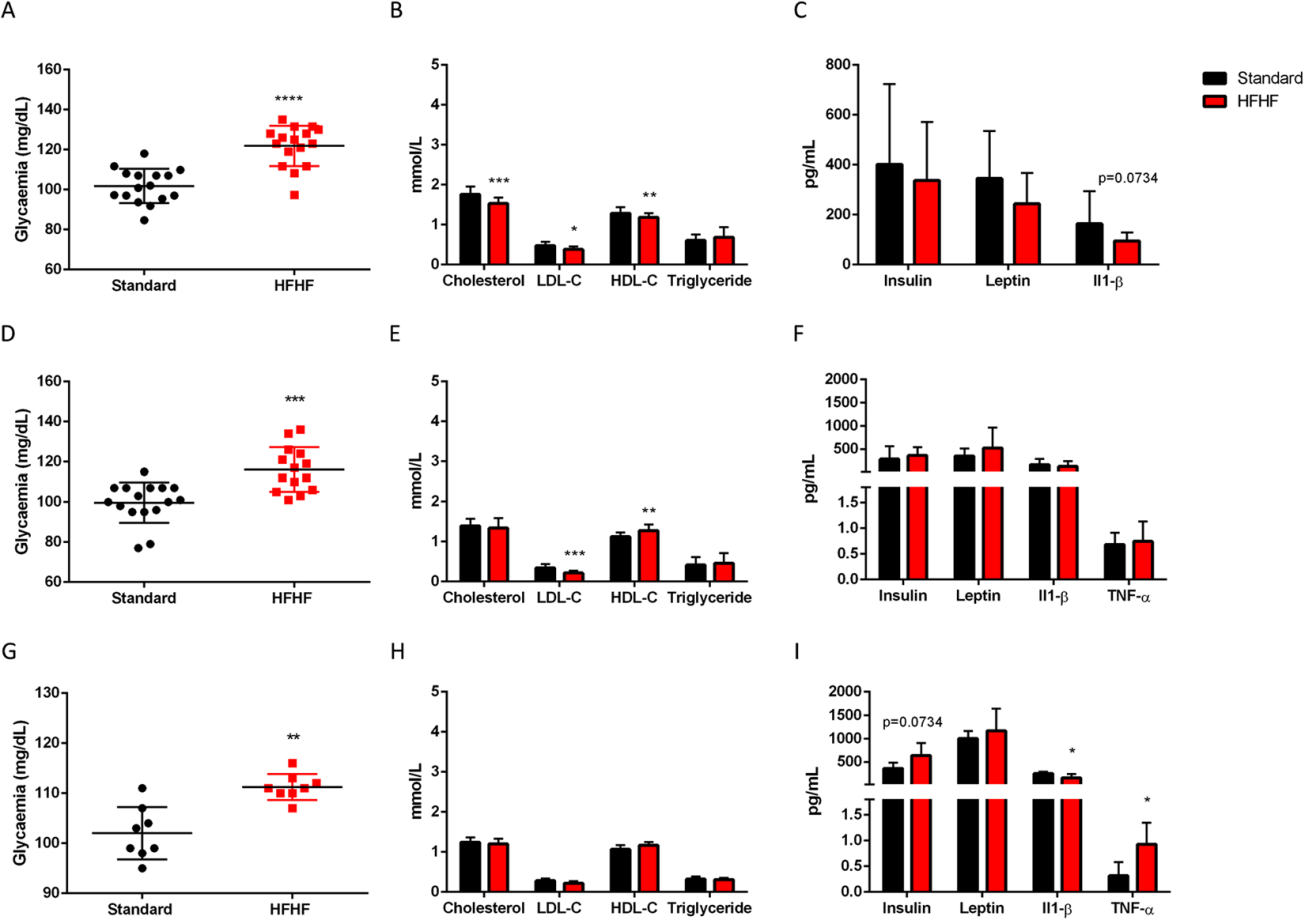
Effect of HFHF diet on plasma analytes. Fasted glycaemia (**A**, for 8 days of diet (n = 16); **D**, for 5 weeks of diet (n = 16) and **G**, for 13 weeks of diet (n = 8)), fasted lipids (mmol/L) (**B**, for 8 days of diet; **E**, for 5 weeks of diet and **H**, for 13 weeks of diet) and fasted insulin, leptine, IL1-β (**C**, for 8 days of diet) and TNF-α (**F**, for 5 weeks of diet and **I**, for 13 weeks of diet). Values are means ± SD (n = 16 for 8 days group, and n = 8 for 5 weeks and 13 weeks). Mann-Whitney test was performed. Data with different * are significantly different at P < 0.05; ** at P < 0.01; *** at P < 0.001; **** at P < 0.0001.

Plasma lipids were quantified at 8 days, 5 weeks, and 13 weeks of diet, as shown in Fig. 2B,E,H. At 8 days of feeding, HFHF diet triggered a decrease in total-cholesterol (P < 0.001), as well as in HDL-cholesterol (HDL-C, P < 0.01) and LDL-cholesterol (LDL-C, P < 0.05). No changes were observed for triglycerides. After 5 weeks of feeding, there was a decrease in LDL-C (P < 0.01) and a significant increase in HDL-C (P < 0.01). On the contrary, no differences were observed between HFHF diet fed rats and standard diet fed rats at 13 weeks.

Cytokines in the plasma were also evaluated at each time-point, shown in Fig. 2C,F,I. Levels of TNFα at 8 days of feeding were under the limit of detection of the luminex sensitivity. There was no difference in the levels of insulin, leptin, IL1β and TNFα at 8 days and 5 weeks of feeding between both diets. Insulinaemia was higher in HFHF diet fed rats at 13 weeks of diet (P = 0.07). Moreover, there was a 2 fold increase of TNFα plasma level (P < 0.05), and a decrease of 36% of IL1β level in HFHF rats compared to rats fed the standard diet at 13 weeks of diet (P < 0.05).

**Figure 3:**
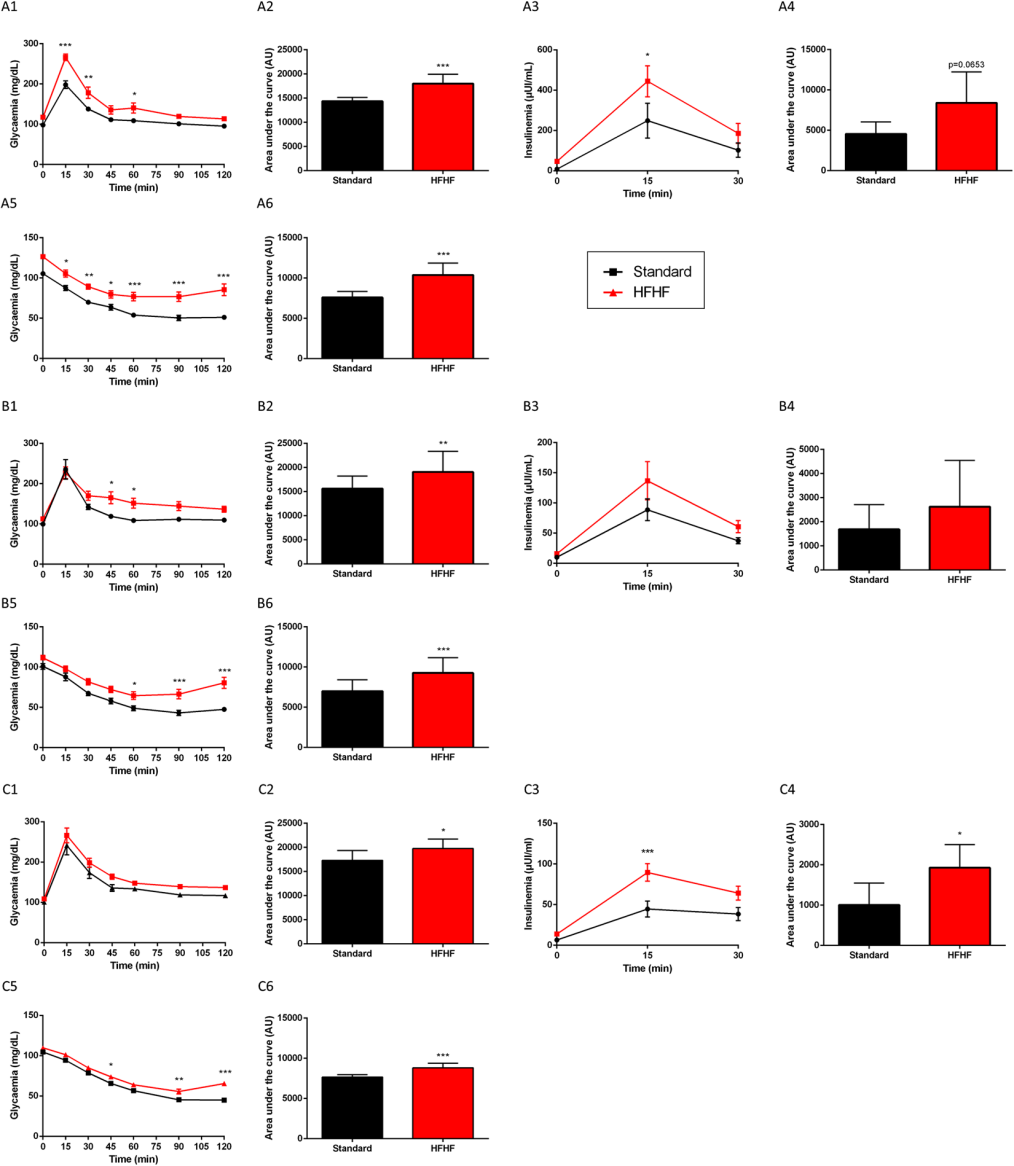
Glucose metabolism is impaired after 8 days of HFHF diet. Blood glucose (A1, for 8 days of diet; B1, for 5 weeks of diet and C1, for 13 weeks of diet) and plasma insulin values (A3, for 8 days of diet; B3, for 5 weeks of diet and C3, for 13 weeks of diet) at different times after intraperitoneal administration of glucose solution (2 g/kg body weight). Area under the curve (AUC) values for glucose (A2, for 8 days of diet; B2, for 5 weeks of diet and C2, for 13 weeks of diet) and insulin (A4, for 8 days of diet; B4, for 5 weeks of diet and C4, for 13 weeks of diet) concentrations. Blood glucose (A5, for 8 days of diet; B5, for 5 weeks of diet and C5, for 13 weeks of diet) values at different times after intraperitoneal administration of an insulin solution (0.5 U/kg body weight). Area under the curve (AUC) values for glucose concentrations (A6, for 8 days of diet; B6, for 5 weeks of diet and C6, for 13 weeks of diet). Results are the mean ± SD (n = 8 animals per group). *P < 0.05; **P < 0.01; ***P < 0.001 after Mann-Whitney and ANOVA test followed by Bonferroni test.

Fasted glycaemia was higher in HFHF diet fed rats vs standard diet fed rats at 8 days (P < 0.0001), 5 weeks (P < 0.001) and 13 weeks (P < 0.01), shown in Fig. 2A,D,G. Intraperitoneal Glucose Tolerance Test (ipGTT) and intraperitoneal Insulin Tolerance Test (ipITT) were performed to evaluate glucose metabolism, as shown in Fig. 3. HFHF induced glucose intolerance at 8 days (P < 0.001), 5 weeks (P < 0.01) and 13 weeks (P < 0.05) of diet, as exemplified by a significantly higher area under the curve (AUC) of plasma glucose in ipGTT (Fig. 3A2,B2,C2). Moreover, HFHF diet fed rats during 13 weeks had higher insulin levels during ipGTT as compared to standard diet fed rats (Fig. 3C3,C4, P < 0.05). HFHF diet induced also insulin resistance as illustrated by higher blood glucose as compared to standard diet fed rats in ipITT. Indeed, HFHF diet fed rats had a significant higher AUC at 8 days (Fig. 3A6, P < 0.001), 5 weeks (Fig. 3B6, P < 0.001) and 13 weeks (Fig. 3C6, P < 0.001) compared to standard diet fed rats.

### HFHF diet induced liver steatosis

**Figure 4:**
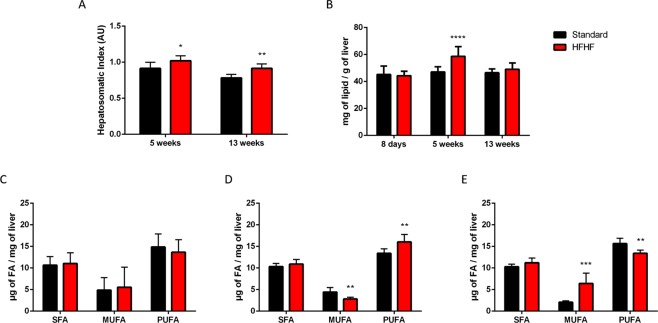
HFHF diet induces liver steatosis. (**A**) Hepatosomatic index. (**B**) Amount of lipids in the liver (milligrams) per gram of liver. Amount of different classes of fatty acids, saturated fatty acids, monounsaturated fatty acids and polyunsaturated fatty acids (micrograms) per milligram of liver of rats fed during 8 days (**C**), 5 weeks (**D**) and 13 weeks (**E**) with HFHF or standard diet. Bars represent the mean ± SD of values obtained from n = 16 (8 days) and n = 8 (5 weeks and 13 weeks). *P < 0.05; **P < 0.01; ***P < 0.001; ****P < 0.0001 after Mann-Whitney test.

The hepatosomatic index was higher in HFHF diet fed rats at 5 weeks (P < 0.05) and 13 weeks (P < 0.01), shown in Fig. 4A. The increase of total lipids in the liver of HFHF diet fed rats during 5 weeks (Fig. 4B, P < 0.0001) suggested the development of liver steatosis. Regarding the quantification of fatty acids in the liver, 5 weeks of HFHF diet induced an increase of polyunsaturated fatty acids (Fig. 4D, P < 0.01) particularly omega-6 classes and on the contrary a decrease of monounsaturated fatty acids (P < 0.01). At 13 weeks of HFHF diet (Fig. 4E), we measured the accumulation of monounsaturated fatty acids (P < 0.001) and depletion in polyunsaturated fatty acids (P < 0.01), both omega-6 and omega-3 classes, hallmarks of liver steatosis.

### HFHF diet induced a reduction of cone sensitivity

#### ERG response

**Figure 5:**
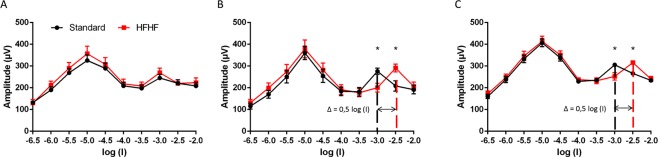
HFHF diet triggers a decrease in cone photoreceptor sensitivity. 8.02 Hz Flicker electroretinographic data of HFHF or standard fed rats during 8 days (**A**), 5 weeks (**B**) and 13 weeks (**C**). Data of the amplitude (µV) of the electroretinographic response as a function of the light stimulus intensity (log(I)), (n = 8 per group). The first peak corresponds to the maximal response of rods and the second peak to the maximal response of cones. Bars represent the mean ± SEM of values obtained from n = 8 per group, *P < 0.05 after ANOVA test.

To assess the functional consequences of HFHF diet on the retina, electrical activity of the retina in response to light was measured by electroretinography (ERG) as described earlier^14^. ERG was recorded after 8 days, 5 weeks and 13 weeks of diet, under dark-adapted scotopic conditions, and light-adapted photopic conditions. ERG were measured after 4 and 5 weeks of diet, and 12 and 13 weeks of diet were compiled and respectively called groups 5 weeks and group 13 weeks. Latencies and amplitudes of both a- and b-waves corresponding to the function of photoreceptors and inner retina respectively were measured. Those parameters were unchanged by HFHF diet at any time, neither in scotopic (Supplementary Tables 1–3) nor in photopic conditions (Supplementary Table 4). Flicker ERG was used to record rod and cone photoreceptors sensitivity to light stimulus at fixed frequency of 8.02 Hz (Supplementary Fig. 2). In Fig. 5, the first peak corresponds to the maximal response of rods and the second peak to the maximal response of cones. Our data showed no differences on the sensibility of rod and cone photoreceptors in rats fed during 8 days with the diets. However, we observed a shift to the right of the maximum response of cone photoreceptors in rats fed with HFHF diet compared to rats fed with standard diet (Δ = 0.5 log(I)), at 5 weeks and 13 weeks, shown in Fig. 5. Altogether, these data suggest a reduction of cone sensitivity in rats fed with HFHF diet compared to standard diet.

### HFHF diet promoted choroidal neovascularization in the retina

**Figure 6:**
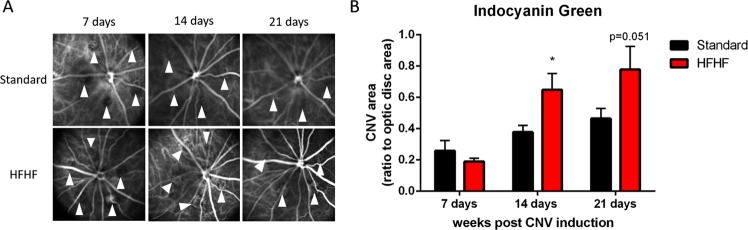
HFHF diet emphasizes laser-induced choroidal neovacularization (CNV). (**A**) Representative images of indocyanine green angiographies taken after 1, 2 and 3 weeks post laser impacts in standard or HFHF Bruch’s membrane. CNV corresponds to the filling of the new vessels as indicated by arrows on the figure. Indocyanine green reveals choroidal vascularization. (**B**) Semi-quantification of CNV (ratio between area of indocyanine green and optic disc area) at 1, 2 and 3 weeks after laser-induced CNV in rats fed during 5 weeks with either the standard or HFHF diets. Bars represent the mean ± SD of values obtained from n = 6. *P < 0.05 after Mann-Whitney test; Standard versus HFHF.

Retinal and choroidal vasculatures were evaluated using cSLO angiography after injection of fluorescein and indocyanine green (ICG) dyes, respectively. Angiography was performed 1, 2 and 3 weeks after the induction of choroidal neovascularization with laser impacts in Bruch’s membrane. Fluorescein corresponds to the retinal vascularization meanwhile indocyanine green reveals choroidal vascularization. Using Brown Norway pigmented rats allowed to make the difference between this both vascular systems. Fluorescein angiography did not show differences between HFHF diet and standard diet (Supplementary Fig. 3). However, ICG angiography revealed an increase of choroidal neovascularization at 2 weeks (Fig. 6, +71%, P < 0.05) and 3 weeks (+67%, P = 0.05) after impacts in Bruch’s membrane in rats fed with HFHF diet (Fig. 6).

### HFHF diet induced retinal gliosis

**Figure 7:**
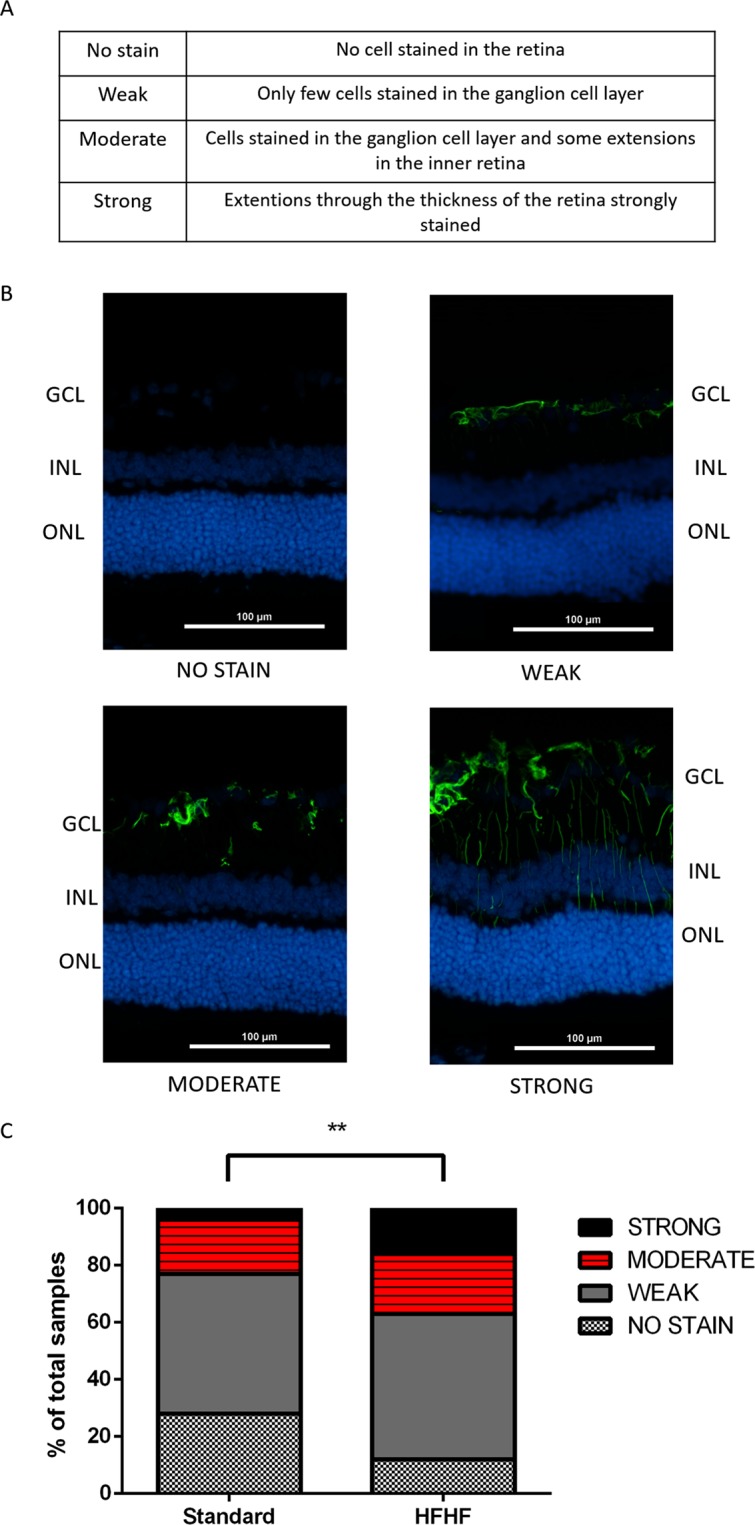
HFHF diet increases retinal gliosis. (**A**) Reading table. (**B**) Representative images of the GFAP IHC signals. (**C**) Double-blind coring of IHC signals associated with GFAP expression in Müller glial cells from strong to no signal. Bar: 100 µm. Values were obtaines from n = 6 eyes per group, 15 sections per eye. Chi-Square test was performed to determine if there was any association between diet and GFAP expression stage; ^**^means P < 0.01. df = 13.74, 3.

The activation of Müller cells was evaluated by semi-quantification of the immunolabeling against the glial fibrillary acidic protein (GFAP). Immunoreactivity was scored from “no stain” to “strong” using a standardized procedure, from retinas of HFHF diet fed rats and Standard fed rats. The results expressed the percentage of the 4 stages of activation of Müller cells in the retina. Blinded scoring revealed higher stain of GFAP cells in HFHF retinas compared to standard retinas (Fig. 7, P < 0.01). The retinas of HFHF fed rats were characterized by a higher effective in the high score suggesting that HFHF diet promoted a pro-inflammatory environment.

## Discussion

Diabetic retinopathy (DR) and Age-related Macular Degeneration (AMD) share common pathogenic pathways such as cellular inflammatory response and oxidative stress. A systematic review and meta-analysis of the literature revealed that diabetes would be a risk factor for the neovascular form of advanced AMD (OR = 1.48 [1.44–1.51, 95% CI] from cross-sectional studies; OR = 1.15 [1.11–1.21, 95% CI] from case-control studies)^16^. Data from the Blue Mountains Eye Study in Australia pointed that obesity, high glucose and high triglyceride were associated with the increased incidence of late AMD during a 10-year follow-up^2^. Most rodent models of diabetes exhibit late events of AMD while the early biomarkers for retinal dysfunction are still lacking^17^. Moreover, retinal abnormalities develop much earlier than the onset of clinical investigation. Thus, a suitable model of insulin resistance and glucose intolerance associated with early retinal dysfunction is needed as potentially valuable tool to investigate the contribution of each risk factor in the development of AMD and DR, in combination with other rat models.

In this study, we provide evidence that an impairment in glucose metabolism, triggered by high fructose and high fat diet (HFHF), is sufficient to induce a reduction of cone sensitivity and to promote choroidal angiogenesis, without the development of dyslipidaemia. Fructose is known to induce MetS characterized by an increase of central fat, a dyslipidaemia, an insulin resistance in rodent models^11,12^, as well as in human^18–20^, and particularly in young adults or children^21,22^. Our results showed that HFHF diet induced fasted hyperglycaemia, glucose intolerance, insulin resistance, very early, i.e. as soon as 8 days after starting feeding, and liver steatosis at 5 and 13 weeks. Moreover, we observed an increase in insulin levels during glucose tolerance test, confirming insulin resistance. Expectedly, fructose did not trigger fasted hyperinsulinaemia, since pancreatic cells lack the fructose transporter GLUT5^23,24^ and fructose bypasses the insulin system. Significant effects on fasted insulinaemia would be expected at later stages of diabetes^25^. Thus, the dysregulation of glucose metabolism seems to be an early phenomenon of the consequences of the HFHF diet in Brown Norway rats. Our model specifically induces hyperglycaemia, glucose intolerance and insulin resistance, which are risk factors for DR and AMD in human.

Among the parameters of the retinal function, cone sensitivity only was significantly reduced by HFHF diet. This finding would be relevant to the effect of diabetes in human vision. Indeed, people with type 1 Diabetes for less than 5 years had subtle impairment of vision, like a loss of blue sensitive color vision without visible abnormalities or retinal changes during a careful ophthalmoscopic examination^26^. Another recent study reported a relationship in color vision impairment in diabetes without retinopathy^27^. This reduction of sensitivity of photoreceptors appears to be an early indicator of neurodegenerative symptoms and that neural damage precedes clinical retinopathy.

In humans, dyslipidaemia, i.e. increase in triglycerides, total cholesterol and LDL-cholesterol, commonly appears while hyperinsulinaemia is observed^28^. Another study by Crapo and collaborators^29^ demonstrated that High-Fructose diet induces hypertriglyceridaemia in diabetics. We hypothesized that the lack of HFHF diet effect on fasted insulinemia could explain why HFHF diet was not effective in inducing dyslipidaemia in Brown Norway rats.

A second cohort of rats was used to investigate the impact of HFHF diet on the vascular status of the retina. Laser impacts on Bruch’s membrane using laser photocoagulation triggers choroidal neovascularization^14,30,31^ has been used extensively in studies related to exudative form of human advanced AMD^31^. Our results showed that HFHF diet during 4 weeks induced an exacerbation of choroidal neovascularization in the retina, 2 and 3 weeks after laser impact on the Bruch’s membrane. This result indicates that HFHF diet promotes a favorable environment for neovascular events. Moreover, chronic hyperglycaemia triggers an early breakdown of the blood retinal barrier, due to RPE dysfunction, then contributing to edema formation, and neovascularization^32^. Müller Glial cells in the retina are the principal glial cells of the retina. These cells are involved in the control of angiogenesis, in neurotrophic functions and by their activation, allows reducing neurotoxicity in destroying neural waste products^33^. This is consistent with our model in which HFHF retinas presented a stronger expression of GFAP compared to standard retinas. This immunoreactivity throughout the retina results from an activation of Müller glial cells. GFAP is the most sensitive early indicator of the activation of glial cells, as soon as there is a retinal injury. Sustained injury induced by impact laser in the Bruch’s membrane leads to Müller glial cells activation at the site of laser impact, in both HFHF retina and standard retina. However, HFHF diet leads to a propagation of this activation, not only at the site of laser impact but also on the rest of the retina, thus reflecting a global inflammation induced by the HFHF diet promoting gliosis and therefore neovascularization. Indeed, mechanisms of this gliosis can be partially explained by *in vitro* studies showing that hyperglycaemia leads to mitochondrial oxidative stress in Müller glial cells and induces radial GFAP expression^34^. Further experiments are necessary to understand the mechanisms and timeframe of HFHF-induced gliosis related to microvascular events in the retina.

The debate is still open to find the origin of the functional changes in the retina during a metabolic syndrome. From our model, we postulate that the deregulation in glucose metabolism would be the key event in the onset of retinal abnormalities, i.e. reduced cone sensitivity. Although these changes are weak, we postulate that deregulations in glucose metabolism are sufficient to create a favorable environment for neovascular and neuronal complications in the retina.

## Materials and Methods

### Animals

**Figure 8:**
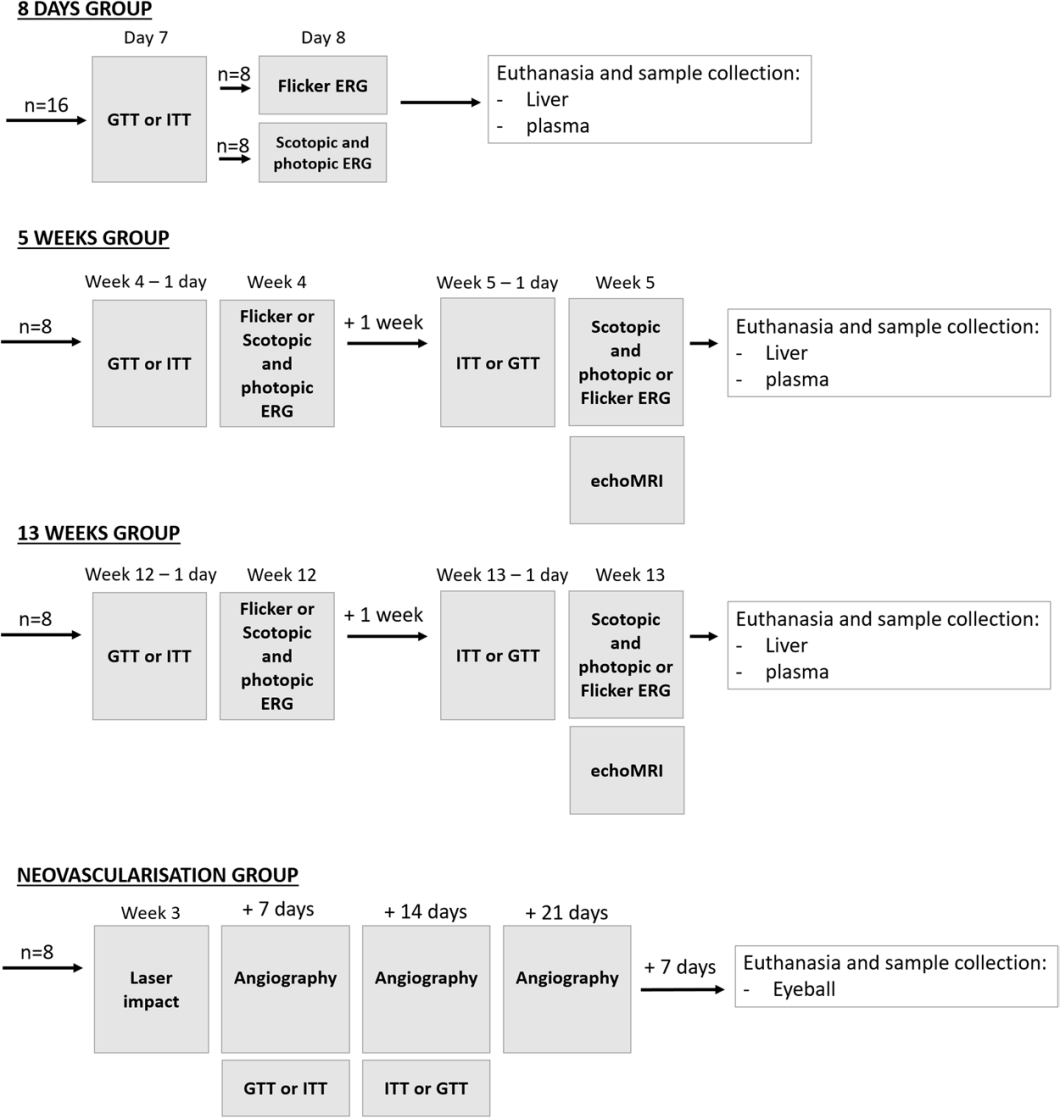
Experimental procedure designed in the goal of reduce number of animal used and refine the procedure. GTT: glucose tolerance test, ITT: insulin tolerance test, ERG: electroretinography.

All animal experiments were conducted in accordance with the ARVO Statement for the Use of Animals in Ophthalmic and Vision Research. The ethics committee of our institution (Comité d’Ethique de l’Expérimentation Animale 105, Dijon, France) approved all interventions and animal care procedures. Male Brown Norway rats (6 weeks of age, n = 80) were purchased from Charles River (L’Arbresle, France). They were housed in a controlled environment at 23 °C ± 1 °C, 55–60% humidity, under a 12-hour light/12-hour dark cycle, 780 lux in light phase, had free access to food and tap water. The rats were acclimatized 2 weeks before they were fed on either a standard rodent chow (Special Diets Services, Witham, UK) or a chow with 60% Fructose and 9% pork lard (HFHF, Sniff Spezialdiäten Gmbh, Soest Germany, Table 1) for 1 week (n = 32), 5 weeks (n = 16) or 13 weeks (n = 16). The experimental design is presented Fig. 8. With ethical concerns, in the goal of reduce number of animal used and refine the procedure, each animal was submitted to intraperitoneal glucose tolerance test (ipGTT) and intraperitoneal insulin tolerance test (ipITT) in a week of interval, as well as photopic and scotopic electroretinography (ERGs) or 8 Hz flicker ERG the next day. All the groups were counterbalanced to avoid any bias linked to the order of experiments, and results were compiled. At 5 and 13 weeks of diet, animals were submitted to body composition analysis by EchoMRI (EchoMRI 500, Houston, Texas; Plateforme de phénotypage du petit animal, Dijon, France).

**Table 1:** Composition of the experimental diets.

Component	Unit	Content	
		Standard	HFHF
Casein	%	14,38	18
Sucrose	%	4,05	0
Fructose	%	0	60
Starch	%	44,97	2,45
Cellulose	%	4,32	4
Soya Oil	%	2,71	0
Soybean Oil	%	0	1
Pork lard	%	0	9
Energy^*^	MJ/kg	14,74	17,2

^*^According to the manufacturer.

Male Brown Norway rats from one other group of animals (6 weeks of age, n = 16) were fed during 3 weeks with the standard diet or the HFHF diet and submitted to argon-laser choroidal neovascularization experiments (see below). This group was submitted to ipGTT and ipITT at 4 and 5 weeks of diet, and the results were compiled with the results of the corresponding group.

### Measurement of intra peritoneal glucose Tolerance Test (ipGTT) and intra peritoneal insulin tolerance test (ipITT)

Rats were placed in individual cages and had free access to tap water. ipGTT and ipITT were performed in 5-h–fasted rats by measuring blood glucose from the tail vein with an automatic blood glucose analyzer (mylife Pura, Ypsomed) before and at 15, 30, 45, 60, 120 minutes after intra peritoneal injection of glucose (2 g/kg) or intraperitoneal injection of insulin (0.5 units/kg). Blood samples were collected for insulin measurements during the ipGTT by using a bead-based AlphaLISA Human Insulin Detection Kit according to manufacturer’s instructions (PerkinElmer).

### Electroretinography

ERGs were recorded after 8 days (n = 32), 4 weeks (n = 16) and 12 weeks (n = 16) of diet, according to ISCEV (International Society for Clinical Electrophysiology of Vision) procedures and using a setup described previously^14^. Rats were dark-adapted (λ < 650 nm) overnight before the experiments and prepared for the ERGs under dim red light. Rats were anesthetized by intraperitoneal injection of ketamine (70 mg/kg body weight; Imalgene 1000, Merial, Lyon, France) and xylazine (14 mg/kg body weight; Rompun 2%, Bayer, Lyon, France) and their pupils were dilated with 1% tropicamide (Mydriaticum, Laboratoire Théa, Clermont-Ferrand, France). Rats were kept on a heat pad during the entire procedure to maintain body temperature at 37 °C. Silver needle electrodes served as reference (forehead) and ground (tail) electrodes, and gold-wire ring electrodes (3 mm) as active electrodes were positioned on the corneal surface of both eyes. Single-flash recordings were obtained under dark-adapted (scotopic) conditions from both eyes simultaneously, after the rats were placed in the Ganzfeld bowl. Stimuli were presented with increasing intensities, reaching from 0.001 cd.s/m^2^ to 10 cd.s/m^2^ divided into 6 steps (0.001, 0.01, 0.1, 1.0, 3.0, 10.0 cd.s/m^2^) with an inter-stimulus interval of 5 seconds and 17 seconds up to 1.0 cd.s/m^2^. The pass-band filter width was 1–300 Hz. Then, light adaptation was performed with a background illumination of 30 cd/m^2^ presented 10 min before recording to obtain cone response after rod saturation and to stabilize photopic responses. Stimuli under light-adapted photopic conditions were presented with increasing intensities, reaching from 0.3 cd.s/m^2^ to 10 cd.s/m^2^, divided into 4 steps (0.3, 1.0, 3.0, 10.0 cd.s/m^2^) with an inter-stimulus interval of 5 seconds and 17 seconds up to 1.0 cd.s/m^2^. The pass-band filter width was 100–500 Hz.

After amplification, the signal was digitized and processed. The amplitudes and latencies of the a- and b-waves were analyzed.

The Flicker ERG examination consisted in fixed frequency 8.02 Hz light stimulation, (n = 8 rats per group). The responses were recorded using ten increasing intensities from 0.0003 cd.s/m^2^ to 10 cd.s/m^2^, with an inter-stimulus interval of 0.126 seconds. The band-pass width was 0.1–3,000 Hz. Recordings were obtained in the morning and early afternoon, and rats from different groups were randomly assigned to avoid bias from circadian variations in responses.

### Argon laser-induced choroidal neovascularization (CNV)

Rats were fed 3 weeks before laser treatment with HFHF diet or Standard diet, and submitted to the procedure of CNV as previously described^14^.

### Funduscopy and angiography

Angiographies were performed 7 days, 14 days and 21 days after laser injury, by confocal scanning laser ophthalmoscopy (cSLO, Heidelberg Engineering, Germany) as previously described^14^.

### Quantification of neovascularization

The analysis of CNV pictures was performed in a double-masked manner by two independent investigators using ImageJ Software as described previously^14^.

### Echo MRI

Body composition was analyzed by quantitative magnetic resonance imaging (EchoMRI 500, EchoMRI Houston, Texas, Plateforme de phénotypage du petit animal, Université de Bourgogne, AgroSup Dijon, France) as described previously^14^, (n = 8 rats per group).

### Collection of samples

Animals were fasted overnight before being euthanized by an intraperitoneal injection of pentobarbital (Ceva, Santé animale, Libourne, France) at lethal dose (150 mg/kg body weight) and exsanguination by intracardiac puncture after 8 days, 5 weeks and 13 weeks of diet, or after 8 weeks of diet in rats with CNV. Plasma was prepared by centrifugation (3000 rpm during 20 min, 4 °C) and kept at −80 °C until further analyses. Liver was weighed, frozen in liquid nitrogen and stored at −80 °C. Eyes and brains were collected for further analyses.

### Plasma analyses

Plasma levels of total cholesterol (TC), high density lipoprotein cholesterol (HDL-C), low density lipoprotein cholesterol (LDL-C), triglycerides (TG) were evaluated by standard automatic analyzers at the Clinical Biochemistry Department of the Dijon University Hospital (Dijon, France). Plasma levels of insulin, leptin, interleukin 1 beta (IL-1β), tumor necrosis factor alpha (TNF-α) were quantified using an adipokine magnetic bead panel (milliplex kit, EMD Millipore Corporation) and assessed by Luminex technology (Biorad Bioplex 200 system, Life Sciences, Marnes-la-Coquette, France) according to the manufacturer’s instructions.

### Liver analyses

Frozen hepatic tissue samples (300 mg) were homogenized in 0.73% of sodium chloride and lipids were extracted following the method of Folch^35^. Fatty acid were converted into methyl esters according to Morrison and Smith^36^ and analyzed as previously described^14^.

### Immunohistochemical labeling and counting of GFAP in the retina

Eyeballs were embedded in Optimal Cutting Temperature (OCT) resin according to the manufacturer’s instructions. Transverse retinal sections (8 µm) were sliced using a cryostat (LEICA 3050 S, Nanterre, France) (n = 6 per group). All retinal sections were blocked for 2 h in a solution containing 10% normal goat serum and 0.1% Triton X-100 in phosphate Buffered Saline (PBS). Primary anti GFAP antibody (sc-6171-R, Santa Cruz Biotechnology, Santa Cruz, California) was diluted in blocking solution (1:500) and incubated at 4 °C overnight. After several washes in PBS, retinal sections were incubated with Alexa fluor goat anti rabbit antiserum (1:500; Jackson Labs, West Grove, PA, United States) diluted in blocking solution for 2 h at room temperature. Sections were also counterstained with DAPI (1:5000) during 2 min. After being washed, tissues were mounted using Vecta Shield (Vector Labs, Burlingame, CA, United States) then analyzed on a Nikon Eclipse E600 upright microscope coupled to a Nikon DS-Ri2 digital camera (NIKON, Champagny sur Marne, France). All the images were obtained in the same conditions of contrast and brightness. Some figures obtained from double-labelling immunofluorescence experiments were processed with ImageJ software. Results from fifteen sections containing the optic nerve were averaged and considered as a single eye result. We analyzed six eyes from six different rats from each group, receiving laser impact. The category of labeling was estimated in double-blind by two different investigators using a score determination. A Chi-Square test was performed to determine if there was any association between diet and GFAP stain stage.

### Statistical analysis

Data were analyzed using the unpaired Mann Whitney test or two way ANOVA followed by a Bonferroni post‐hoc test when appropriate. The level of significance was set at P ≤ 0.05. All results were analyzed using GraphPad Prism version 7.00 for Windows (GraphPad Software, San Diego, CA, United States). Data are presented as mean ± standard deviation (SD) or standard error of the mean (SEM) when appropriate.

## Supplementary information

Supplementary figures and tables
