# Supplementary material for: Early impairments in the retina of rats fed with high fructose/high fat diet are associated with glucose metabolism deregulation but not dyslipidaemia
Source: Sci Rep. 2019 Apr 12;9:5997. doi: 10.1038/s41598-019-42528-9 (PMC6461688; doi:10.1038/s41598-019-42528-9)
Supplement: Supplementary file 1 — Supplementary figures and tables [file 41598_2019_42528_MOESM1_ESM.pdf]

Supplementary information

Early impairments in the retina of rats fed with high fructose/high fat diet are associated with glucose metabolism deregulation but not dyslipidaemia

Elisa Vidal[1, 2], Elise Lalarme[3], Marie-Annick Maire[1], Valérie Febvret[1], Stéphane Grégoire[1], Ségolène Gambert[1, 4], Niyazi Acar[1], Lionel Bretillon[1*]

[1] Eye and Nutrition Research Group, Centre des Sciences du Goût et de l'Alimentation, AgroSup Dijon, INRA, CNRS, Université Bourgogne Franche-Comté, Dijon, France

[2] Horus Pharma Laboratories, Saint Laurent du Var, France

[3] Animalerie Expérimentale, Centre des Sciences du Goût et de l'Alimentation, AgroSup Dijon, INRA, CNRS, Université Bourgogne Franche-Comté, Dijon, France

[4] Department of Clinical Chemistry, University Hospital, Dijon, France

Supplementary Figure 1. Food (A) and water (B) consumption (grams) per rat during the 13 weeks of nutritional experiments. Values are means ± SEM. ANOVA followed by Bonferroni test was performed. **: P < 0.01 and ****: P < 0.0001.

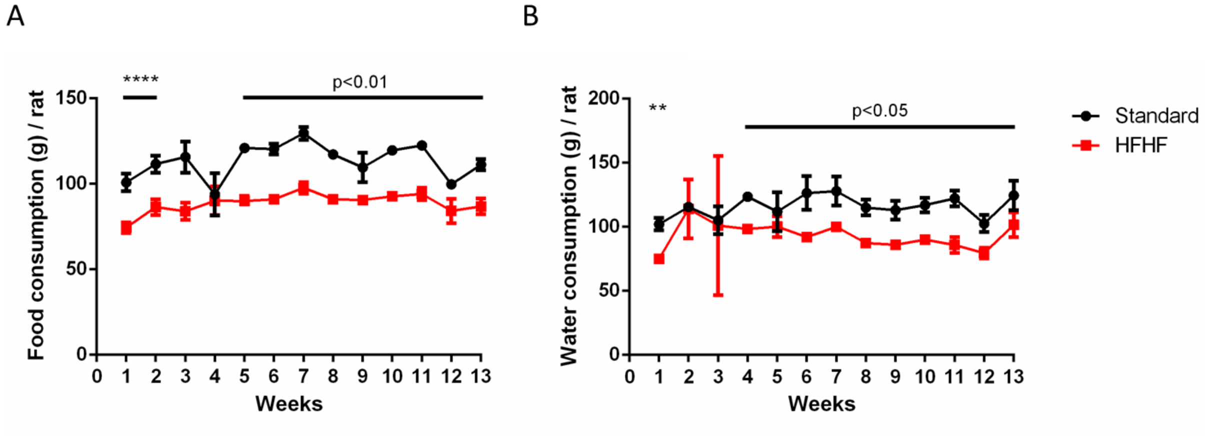

Supplementary Figure 2. Waveforms from flicker ERG of retina of standard and HFHF fed rats. Flicker ERG waveform of rats fed for 8 days (A and B), 5 weeks (C and D) and 13 weeks (D and E) with standard or HFHF diet respectively.

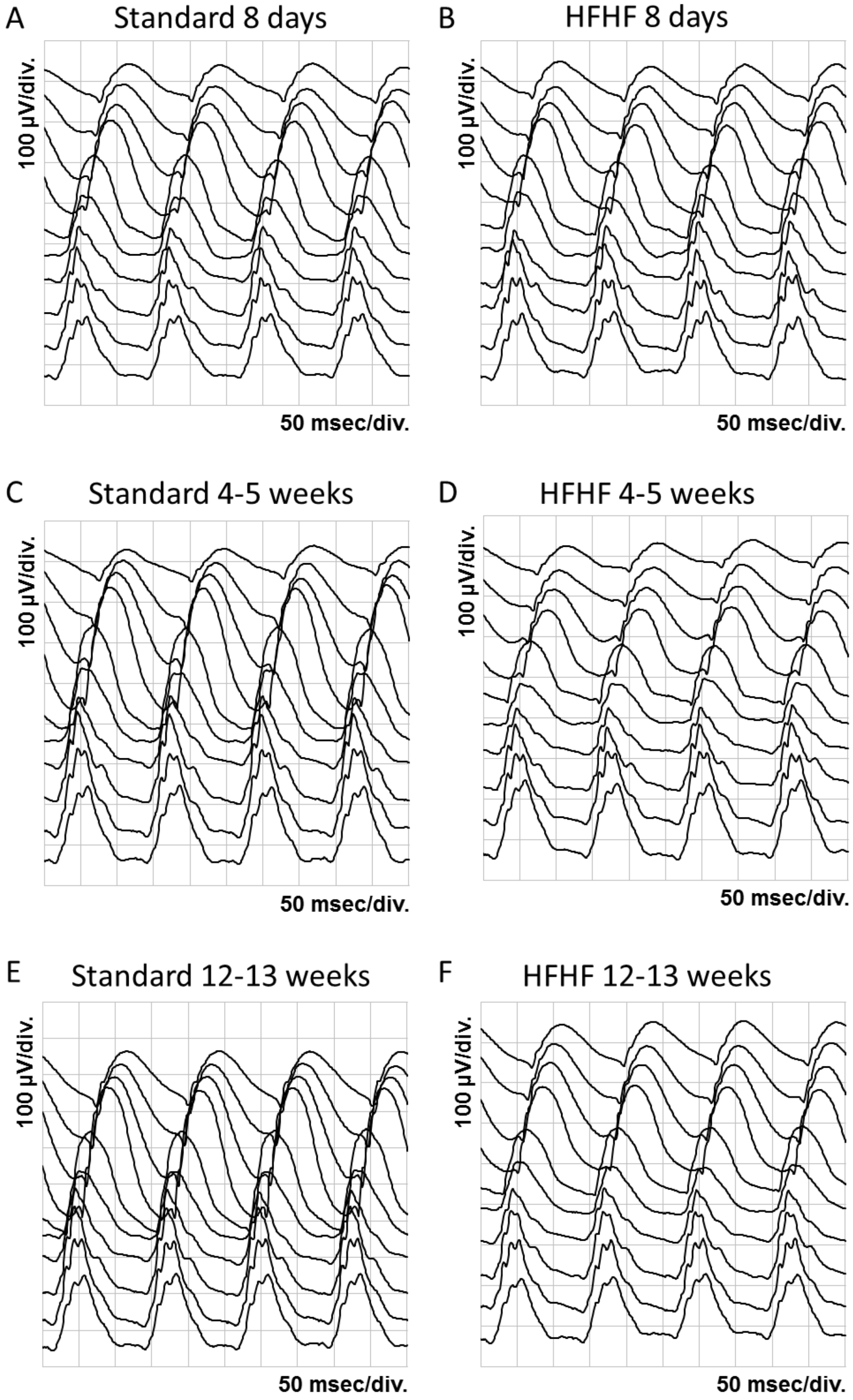

Supplementary Figure 3. (A) Representative images of fluorescein angiographies taken after 1, 2 and 3 weeks post laser impacts in standard or HFHF Bruch's membrane. CNV correspond to the filling of the new vessels. Fluorescein corresponds to the retinal vascularisation meanwhile indocyanin green reveals choroidal vascularisation. Using Brown Norway pigmented rats allowed to make the difference between this both vascular systems. (B) Semi-quantification of CNV (ratio between area of fluorescein and optic disc area) at 1, 2 and 3 weeks after laser-induced CNV in rats fed during 5 weeks with either the standard or HFHF diets. Bars represent the mean ± SD of values obtained from n = 6. *: P < 0.05; Standard versus HFHF (Mann-Whitney test).

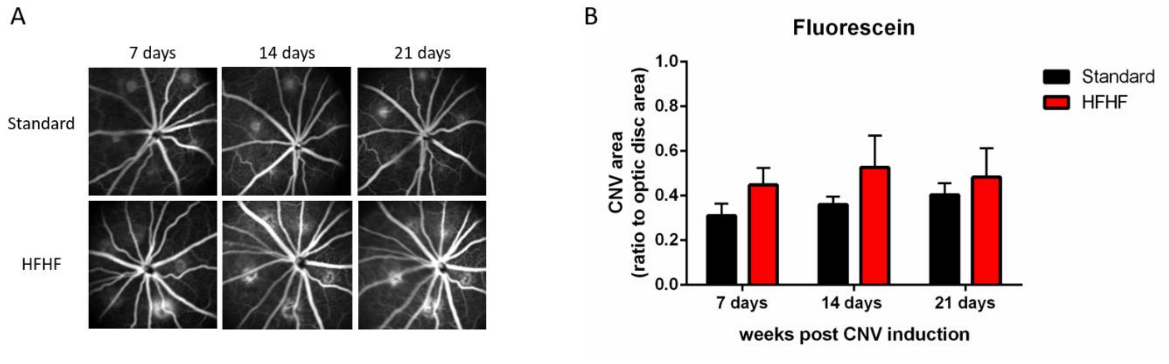

Supplementary Table 1. Measure of scotopic electroretinogram of HFHF and Standard-fed rats during 8 days. (A) Scotopic b-wave amplitude. (B) Scotopic b-wave latency. (C) Scotopic a-wave amplitude. (D) Scotopic a-wave latency SD, standard deviation.

A

Light intensity (cds/m²)		0.001	0.01	0.1	1	3	10
HFHF	Mean	300.3	491.6	484.6	676.8	793.3	791.9
	SD	102.7	162.15	150.74	253.36	290.13	310.7
Standard	Mean	285.3	508.7	488.9	699.8	806.4	804.1
	SD	83.25	155.47	164.63	266.85	298.89	297.6

B

Light intensity (cds/m²)		0.001	0.01	0.1	1	3	10
HFHF	Mean	78.9	58.2	42.5	43.8	45.3	43.0
	SD	3.3941	9.0527	2.0475	4.6167	5.3689	6.3557
Standard	Mean	80.6	61.7	41.3	41.4	47.2	44.8
	SD	4.5547	3.9421	2.1731	4.7932	4.9791	5.7114

C

Light intensity (cds/m²)		0.1	1	3	10
HFHF	Mean	98.2	301.9	361.3	426.9
	SD	37.387	113.22	142.07	159.51
Standard	Mean	93.5	306.6	362.6	415.7
	SD	42.139	124.58	147.75	160.39

D

Light intensity (cds/m²)		0.1	1	3	10
HFHF	Mean	22.1	18.8	13.2	11.8
	SD	0.4351	0.9777	0.3225	0.2733
Standard	Mean	22.2	19.7	13.5	12.3
	SD	0.7572	0.6831	0.3225	0.4817

Supplementary Table 2. Measure of scotopic electroretinogram of HFHF and Standard-fed rats during 5 weeks. (A) Scotopic b-wave amplitude. (B) Scotopic b-wave latency. (C) Scotopic a-wave amplitude. (D) Scotopic a-wave latency SD, standard deviation.

A

Light intensity (cds/m^2)		0.001	0.01	0.1	1	3	10
HFHF	Mean	363.8	617.6	565.6	866.5	983.3	1020.1
	SD	76.029	130.62	153.85	251.59	295.95	268.58
Standard	Mean	373.1	560.1	566.5	865.0	997.6	1061.6
	SD	123.58	142.75	182.39	304.29	307.17	295.13

B

Light intensity (cds/m^2)		0.001	0.01	0.1	1	3	10
HFHF	Mean	82.0	61.4	43.7	48.3	50.6	49.3
	SD	3.802	3.7302	2.6894	3.1031	2.0682	3.2745
Standard	Mean	84.8	63.5	46.2	49.7	53.0	52.7
	SD	3.507	3.0068	1.6211	2.0454	4.8708	3.8668

C

Light intensity (cds/m^2)		0.1	1	3	10
HFHF	Mean	124.5	374.6	465.3	540.8
	SD	33.716	111.11	136.79	150.42
Standard	Mean	119.5	359.2	462.1	531.5
	SD	35.929	112.9	134.72	148.74

D

Light intensity (cds/m^2)		0.1	1	3	10
HFHF	Mean	22.1	18.9	13.3	11.8
	SD	0.5241	0.575	0.4161	0.3386
Standard	Mean	22.4	19.3	13.3	11.9
	SD	0.7137	1.099	0.354	0.3225

Supplementary Table 3. Measure of scotopic electroretinogram of HFHF and Standard-fed rats during 13 weeks. (A) Scotopic b-wave amplitude. (B) Scotopic b-wave latency. (C) Scotopic a-wave amplitude. (D) Scotopic a-wave latency SD, standard deviation.

A

Light intensity (cds/m^2)		0.001	0.01	0.1	1	3	10
HFHF	Mean	443.6	646.3	679.5	1032.7	1161.0	1132.9
	SD	153.35	183.66	199.26	270.08	267.46	246.41
Standard	Mean	370.6	581.7	533.5	838.3	947.4	980.4
	SD	111.4	170.9	162.23	236.02	270.79	242.85

B

Light intensity (cds/m^2)		0.001	0.01	0.1	1	3	10
HFHF	Mean	86.6	63.3	44.5	50.4	52.1	49.5
	SD	3.6124	3.852	2.8896	1.9541	2.601	1.1764
Standard	Mean	84.9	62.7	43.2	49.8	50.8	50.4
	SD	2.0971	3.3995	3.1898	2.557	2.4063	1.5448

C

Light intensity (cds/m^2)		0.1	1	3	10
HFHF	Mean	132.9	413.4	523.2	579.9
	SD	41.37	111.26	125	135.13
Standard	Mean	111.6	337.1	431.4	485.8
	SD	28.959	99.397	108.51	114.19

D

Light intensity (cds/m^2)		0.1	1	3	10
HFHF	Mean	22.3	19.3	13.4	12.0
	SD	0.6532	0.7711	0.459	0.4
Standard	Mean	22.5	19.0	13.3	11.9
	SD	0.3578	1.481	0.4131	0.3578

Supplementary Table 4. Measure of photopic electroretinogram of HFHF and Standard-fed rats during 8 days (A and B), 5 weeks (C and D) and 13 weeks (E and F). (A) Photopic b-wave amplitude for 8 days. (B) Photopic b-wave latency for 8 days. (C) Photopic b-wave amplitude for 5 weeks. (D) Photopic b-wave latency for 5 weeks. (E) Photopic b-wave amplitude for 13 weeks. (F) Photopic b-wave latency for 13 weeks. SD, standard deviation.

A

Light intensity (cds/m^2)		0.3	1	3	10
HFHF	Mean	4.8	8.3	21.0	134.6
	SD	2.2948	3.2153	5.1832	26.911
Standard	Mean	5.1	4.1	23.4	144.9
	SD	2.354	3.4787	8.2441	40.953

B

Light intensity (cds/m^2)		0.3	1	3	10
HFHF	Mean	29.9	42.8	39.1	50.4
	SD	2.4511	1.2699	1.0854	5.7709
Standard	Mean	30.0	41.5	38.4	49.9
	SD	1.7761	1.7293	0.8512	4.7574

C

Light intensity (cds/m^2)		0.3	1	3	10
HFHF	Mean	7.5	5.8	30.0	160.5
	SD	2.6572	2.357	5.9166	27.166
Standard	Mean	5.7	6.0	27.7	151.6
	SD	1.7914	3.6733	8.8157	39.694

D

Light intensity (cds/m^2)		0.3	1	3	10
HFHF	Mean	31.0	43.1	39.4	44.3
	SD	1.2212	1.8437	0.8991	1.2056
Standard	Mean	31.2	43.3	39.9	45.0
	SD	1.2454	1.8435	0.935	1.2625

E

Light intensity (cds/m^2)		0.3	1	3	10
HFHF	Mean	6.1	5.6	30.6	176.9
	SD	2.5123	2.5323	6.834	28.917
Standard	Mean	5.7	5.1	26.1	153.0
	SD	1.9766	2.807	7.0938	34.736

F

Light intensity (cds/m^2)		0.3	1	3	10
HFHF	Mean	31.1	42.9	39.4	45.8
	SD	0.7561	1.1213	0.8805	2.1875
Standard	Mean	31.0	43.3	39.8	46.1
	SD	1.7826	1.0274	0.5987	2.7808
